# Rapid Diagnosis of Recurrent Paucibacillary Tuberculosis

**DOI:** 10.20411/pai.v7i2.565

**Published:** 2023-04-19

**Authors:** Claudia Jafari, Ioana D. Olaru, Franziska Daduna, Christoph Lange, Barbara Kalsdorf

**Affiliations:** 1 Division of Clinical Infectious Diseases, Research Center Borstel, Borstel, Germany; 2 London School of Hygiene and Tropical Medicine, London, United Kingdom; 3 Institute of Medical Microbiology, University of Münster, Münster, Germany; 4 Respiratory Medicine & International Health, University of Lübeck, Lübeck, Germany; 5 German Center for Infection Research (DZIF), Partner site Hamburg-Lübeck-Borstel, Germany; 6 Baylor College of Medicine and Texas Children's Hospital, Houston, Texas

**Keywords:** BAL, ELISPOT, GeneXpert, pulmonary TB

## Abstract

**Introduction:**

The rapid diagnosis of tuberculosis recurrence can be challenging due to persistently positive detection of *Mycobacterium tuberculosis*-specific DNA from sputum and bronchopulmonary samples in the absence of active disease.

**Methods:**

We compared the diagnostic accuracy of the detection of *M. tuberculosis*-specific DNA by either Xpert (January 2010-June 2018) or Xpert Ultra (July 2018-June 2020) and *M. tuberculosis*-specific ELISPOT in bronchoalveolar lavage (BAL) samples with *M. tuberculosis* culture results from sputum or bronchopulmonary samples in patients with suspected recurrence of pulmonary tuberculosis.

**Results:**

Among 44 individuals with previous tuberculosis and a presumptive diagnosis of recurrent pulmonary tuberculosis, 4/44 (9.1%) were diagnosed with recurrent tuberculosis by culture. DNA of *M. tuberculosis* was detected by Xpert in BAL fluid in 1/4 (25%) individuals with recurrent tuberculosis and in 2/40 (5%) cases with past tuberculosis without recurrence, while BAL-ELISPOT with a cut-off of >4,000 early secretory antigenic target-6-specific or culture filtrate protein-10-specific interferon-γ-producing lymphocytes per 1 million BAL-lymphocytes was positive in 4/4 (100%) individuals with recurrent tuberculosis and in 2/40 (5%) cases of past tuberculosis without recurrence.

**Conclusion:**

*M. tuberculosis*-specific BAL-ELISPOT is more accurate than BAL-Xpert for the diagnosis of paucibacillary tuberculosis recurrence.

## INTRODUCTION

Early diagnosis of paucibacillary pulmonary tuberculosis is crucial to ensure early treatment as well as avert disease progression and transmission. A positive culture result for *Mycobacterium tuberculosis* remains the gold standard, but although culture techniques have improved [1], the turnaround times of cultures are long – with 2-6 weeks for positive and up to 8 weeks for negative cultures [2, 3]. Among the diagnostic technologies endorsed by the WHO are Xpert MTB/RIF and Xpert Ultra (Cepheid, Sunnyvale, CA) as initial diagnostic tests [4]. Both have excellent sensitivities and specificities of 85% and 99% for Xpert MTB/RIF and 90% and 96% for Xpert Ultra, respectively [4]. However sensitivity decreases to less than 70% in patients with tuberculosis and undetectable acid-fast bacilli on sputum microscopy who represent over 50% of all patients with tuberculosis [5]. Interferon-γ release assays (IGRA) are not as easy to perform and are more expensive. Routinely, IGRAs are performed on cells derived from peripheral blood to document *M. tuberculosis* antigen exposure as they cannot distinguish between active tuberculosis, latent infection with *M. tuberculosis* (LTBI) or past disease [6–8]. Therefore, IGRAs are not recommended by the WHO for diagnosis of active tuberculosis in low and middle income countries [9]. *M. tuberculosis*-specific enzyme-linked immunospot (ELISPOT)-IGRA has a high sensitivity to diagnose tuberculosis in patients with undetectable acid-fast bacilli on sputum smear microscopy if performed on specimens from the site of the disease (eg, bronchoalveolar lavage fluid (BAL), pleural fluid, ascites, or cerebrospinal fluid) because effector T-cells clonally expand and are enriched at the site of the infection [10–14]. In a previous study, we showed the high accuracy of >95% of a stepwise diagnostic algorithm for the rapid diagnosis of pulmonary tuberculosis by BAL-GeneX-pert followed by *M. tuberculosis*-specific ELISPOT-IGRA from BAL [15].

According to the WHO, an estimated 7.7% of all patients with tuberculosis had experienced a previous tuberculosis episode [16]. In Germany, a country with a low tuberculosis incidence, almost 10% of patients presenting with a new episode of tuberculosis had a previous diagnosis of tuberculosis [17]. Recurrent tuberculosis causes further lung damage [18] and is associated with an increased risk of drug resistance [19, 20]. In cases of tuberculosis recurrence, the performance of IGRA and Xpert tests remains unclear. Because of persisting mycobacterial DNA, patients with respiratory symptoms who had experienced a previous tuberculosis episode may have positive Xpert results in the absence of recurrent disease leading to unnecessary treatment [21] [22]. In a large cohort of pretreated patients with symptoms of recurrent tuberculosis, 1 in 7 (14.3%) patients had a positive Xpert result without recurrent disease and received unnecessary retreatment [23]. Cases of persistent positive GeneXpert results were reported up to more than 7 years after treatment completion [24, 25]. Blood immune responses, for example, positive peripheral blood mononuclear cell (PBMC)-ELISPOT results variably persist after treatment and recovery from tuberculosis [26, 27].

The aim of the present study was to investigate the diagnostic accuracy of BAL-Xpert and BAL-ELISPOT with the goal of early identification and treatment of patients with recurrent tuberculosis and to avoid unnecessary treatment in patients with a history of tuberculosis without recurrence.

## METHODS

### Study Participants

Patients were prospectively enrolled from January 2010 to June 2020 at the Medical Clinic of the Research Center Borstel, the site of the National Reference Center for tuberculosis in Germany. All patients (≥ 16 years) referred to the hospital with symptoms suggestive of TB (≥1 of the following: cough, fever, night sweats, and/or weight loss), radiological signs suggestive of tuberculosis on chest x-ray or computed tomography, and undetectable acid-fast bacilli (AFBs) on sputum smear microscopy underwent routine diagnostic procedures. These included bronchoscopy with BAL from a radiographically abnormal lung area including BAL-ELISPOT. All respiratory samples were evaluated by *M. tuberculosis* culture. Patients with previous tuberculosis treatment and clinically suspected recurrence were included in the study. Active tuberculosis was defined as at least 1 positive culture for *M. tuberculosis.* A subgroup of the patients (n=2 with active tuberculosis and n=5 with previous tuberculosis diagnosis and no recurrence) enrolled between November 2011 and June 2016, have been included in a previous publication [15]. All patient data were anonymized and reporting follows the STARD criteria [28].

### Sputum Microscopy

Sputum microscopy was performed using Ziehl-Neelsen staining with light microscopy (January 2010-July 2017) and auramine staining with fluorescence microscopy (August 2017-June 2020).

### *M. tuberculosis* Culture

For each respiratory sample obtained, 1 liquid culture (BD BACTEC MGIT system) and 2 solid cultures (Loewenstein-Jensen medium and Stonebrink medium) were prepared.

### *M. tuberculosis*-specific ELISPOT

*M. tuberculosis*-specific ELISPOT was performed on mononuclear cells derived from peripheral blood or BAL as described previously [10]. The assay was performed with test plates from the T-SPOT.TB test (Oxford Immunotec, Abingdon, UK). Incubation of the plates, washing, counter-staining, visualization, and analysis of the spots were performed according to the manufacturer's instructions [29]. Results of *M. tuberculosis*-specific ELISPOT on PBMC were considered positive, if 5 or more spot-forming cells (SFCs) were observed in the early secretory antigenic target (ESAT)-6 or the culture filtrate protein (CFP)-10 well, after subtracting the number of SFCs in the negative control well. Results of *M. tuberculosis*-specific ELISPOT on BAL were considered positive if more than 5 SFCs were observed in the ESAT-6 or the CFP-10 well, after subtracting the number of SFCs in the negative control well, and if the total number of SFCs in the ESAT-6 or CFP-10 well was at least twice the number of SFCs in the negative control well. The results were considered negative if they did not meet the definition for a positive result, and if the number of SFCs in the positive control was >20 SFCs after subtracting the number of SFCs in the negative control well, and if the positive control had at least twice the number of SFCs of the negative control well. Indeterminate results were defined as not meeting the criteria for a positive or a negative test result [11]. GeneXpert results were not known to the technician performing the ELISPOT tests.

### Determination of Lymphocyte Number in BAL by FACS Analysis

To adjust for the different percentages of BAL-lymphocytes in the individual patient, BALCs were acquired on a FACSCalibur flow cytometer (BD Bioscience, Heidelberg, Germany). BAL-monocytes/macrophages (Region 1) and BAL-lymphocytes (Region 2) were identified by forward scatter (FSC) height versus side scatter (SSC) height, (Supplementary Figure 1). The respective percentage of BAL-lymphocytes out of the population of BAL-monocyte/macrophages (R1) and BAL-lymphocytes (R2) was used to extrapolate SFCs per 250,000 cells per well to 1 million lymphocytes in BAL as previously described [15].

**Calculation:** (SFC / 250,000 BALCs X 4) x (G1+G2 in %) / (G2 in %) = SFC / 1 million BAL-lymphocytes

### Interpretation of BAL-ELISPOT Results

1. Conventional cut-off: more than 5 *M. tuberculosis*-specific SFC / 250,000 mononuclear cells. This was used for an initial BAL-ELISPOT analysis to determine whether the test was positive or negative [11].
2. To optimize BAL-ELISPOT performance for the diagnosis of active pulmonary tuberculosis [10, 11], new interpretation strategies were investigated recently [15, 30]. In a comparison of different interpretation strategies comprising 100 patients with BAL-ELISPOT results, our group proposed to normalize ESAT-6 and CPF 10 SFC / 250,000 BAL cells for the lymphocyte number measured by FACS analysis and express results as ESAT-6 or CFP 10 SFC / 1 million lymphocytes in BAL fluid. This strategy performed with the highest sensitivity (89%) and specificity (97%) of all those included in the previous study [15] as it accounts for the variable number of lymphocytes found in BAL. In the present study, this previously described interpretation strategy was applied.

### Xpert

Xpert MTB/RIF (January 2010-June 2018) and Xpert Ultra (July 2018-June 2020) were performed according to manufacturer's instructions [31] at the National Reference Center for Mycobacteria in Borstel, Germany on sputum and BAL samples.

### Statistical Analysis

Categorical variables were reported using counts and percentages and continuous variables using medians and interquartile ranges (IQR). For the test performance analysis, the reference standard was considered *M. tuberculosis* culture positivity. Differences between groups were evaluated using the chi-square test for categorical variables and the Wilcoxon rank sum test for continuous variables. Statistical analysis was performed using R v4.0.3 (The R Foundation for Statistical Computing, Vienna, Austria).

### Ethics

The study was approved by the Ethical committee of the University of Lübeck (14-031A). Informed consent was not necessary because only anonymized routine clinical data were analyzed retrospectively.

## RESULTS

### Patient Characteristics

**Figure 1.**
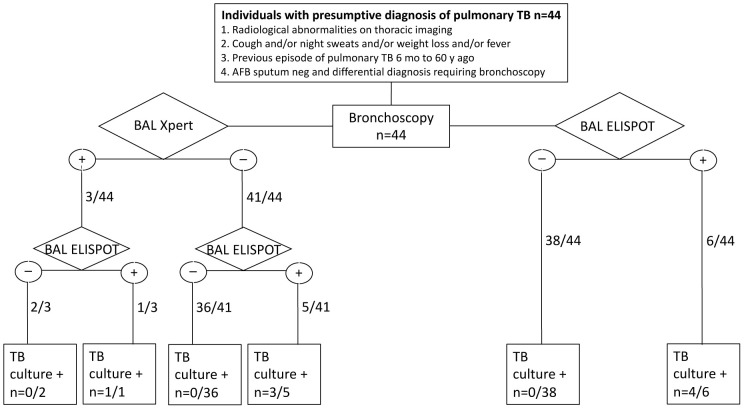
Study flow diagram of individuals with a presumptive diagnosis of pulmonary tuberculosis (n=44). Patients with clinical signs of infection, radiological abnormalities on thoracic imaging, a previous episode of pulmonary tuberculosis, negative microscopy for AFB on sputum, and a differential diagnosis requiring bronchoscopy were tested for BAL Xpert, BAL Elispot, and TB culture results. AFB: acid-fast bacilli, BAL: bronchoalveolar lavage.

Between January 2010 and June 2020, there were 352 individuals with clinically suspected active pulmonary tuberculosis with undetectable acid-fast bacilli on sputum smear microscopy who underwent bronchoscopy. Of those, 308/352 (87.5%) were excluded due to ineligibility: 24 had a first episode of active tuberculosis, 67 were diagnosed with latent infection with *M. tuberculosis* (LTBI), and 163 had a different final diagnosis other than tuberculosis (Figure 1). Among the 44 individuals with a history of tuberculosis, the previous tuberculosis episode was reported between 6 months to 60 years prior to the current hospital admission. Of these, 11/44 had a previous episode of tuberculosis less than 2 years prior. Recurrent tuberculosis was diagnosed in 4/44 (9.1%) by detectable *M. tuberculosis* growth in culture (Figure 1). Among the remaining 40 individuals with a prior history of tuberculosis and suspected recurrence, active disease was excluded by at least 3 negative culture results for *M. tuberculosis* from the current hospital admission and no positive culture on follow-up. The median age was 51 years (IQR 41-68 years) and 29 (66%) were male. One individual was HIV-positive.

### PBMC-ELISPOT Results

Among 44 individuals with prior tuberculosis, 36 had a positive PBMC-ELISPOT result, this includes all patients diagnosed with active TB (4/4) and 32/40 (80%) individuals with previous TB and without recurrence.

### Xpert Results from BAL

**Figure 2.**
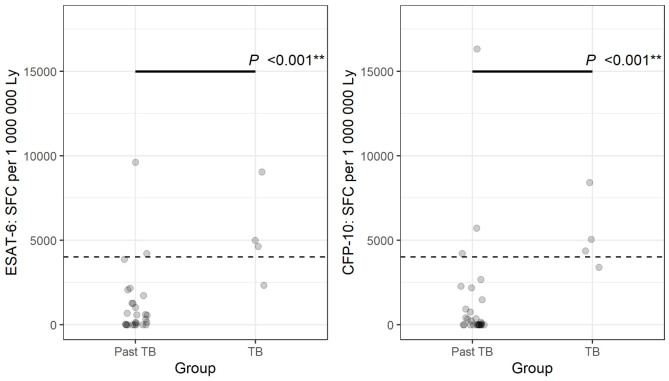
Number of spot-forming cells (SFCs) for early secretory antigenic target (ESAT)-6 and culture filtrate protein (CFP) 10 specific bronchoalveolar lavage cells per 1 million lymphocytes in 44 individuals with a prior tuberculosis episode and recurrence and those without recurrence. All 4 patients diagnosed with active tuberculosis had more than 4000 SFC after stimulation with either ESAT-6 or CFP 10 or both interpreted as a positive result.

*NOTE: Although for each of the individual measurements of ESAT-6 and CFP-10, there was 1 patient in the TB group who had fewer than 4000 SFC per 1 million lymphocytes, all 4 patients had either 1 or the other measurement above the given threshold (see values in Table 1).*

Out of 44 individuals with a prior tuberculosis episode, 3 had a positive BAL-Xpert result. Only 1 patient had a positive culture result for *M. tuberculosis,* while in the other 2, recurrent tuberculosis was excluded by negative culture results and absence of clinical diagnosis of tuberculosis during follow-up (Table 1, Figure 2, *P*=0.757).

**Table 1.** Characteristics of Patients with 1 or More Positive Tests (>4,000 BAL lymphocytes) (ELISPOT, BAL-GeneXpert, culture).

**A**											
Patient	Age in years	Sex	HIV	Time between first TB and reinvestigation in years	Country of origin	MTB culture	AFB BAL	Xpert BAL	PBMC Elispot	BAL Elispot	TTP in days
1	52.7	m	−	25	Macedonia	+	−	+	+	+	33
2	30.6	m	−	17	Sudan	+	−	−	+	+	17
3	64.2	m	−	50	Afghanistan	+	−	−	+	+	34
4	48.7	m	−	2	Russia	+	−	−	+	+	26
5	47.2	m	+	1.5	Ukraine	−	−	+	−	−	−
6	77.5	f	−	1.7	Germany	−	−	+	+	−	−
7	43.2	f	−	2	Croatia	−	−	−	+	+	−
8	37.9	m	−	6	India	−	−	−	+	+	−

B						
Patient	SFC per 250.000 cells				SFC per 1.000.000 lymphocytes	
	PBMC	PBMC	BAL	BAL	BAL	BAL
	ESAT-6	CFP-10	ESAT-6	CFP-10	ESAT-6	CFP-10
1	15*	12*	50*	34*	5000*	3400
2	473*	301*	407*	379*	9044*	8422*
3	91*	40*	79*	170*	2346	5048*
4	16*	23*	53*	50*	4639*	4376*
5	2	1	3	9*	141	424
6	24*	40*	0	0	0	0
7	25*	32*	149*	253*	9613*	16323*
8	2	8*	45*	45*	4215*	4215*

TB: tuberculosis, MTB: *Mycobacterium tuberculosis*, AFB: acid-fast bacilli, BAL: bronchoalveolar lavage, PBMC: peripheral blood mononuclear cells, TTP: time to culture positivity, SFC: spot-forming cells, ESAT-6: Early secretory antigenic target 6kDa. CFP 10: culture filtrate protein 10.

A) Individual test results and patient characteristics of the 8 patients

B) Individual ELISPOT results for the 8 patients;

* indicates a positive test result

### BAL-ELISPOT Results

Using the conventional ELISPOT cut-off (5 or more ESAT-6 or CFP 10 specific cells / 250,000 BAL cells), 17/44 individuals with a prior tuberculosis diagnosis had a positive result in the BAL: 4/4 with recurrent tuberculosis and 13/40 (32.5%) with no recurrence (*P* = 0.035).

Using the cut-off of >4,000 ESAT-6 or CFP 10 specific cells / 1 million BAL lymphocytes, 6/44 individuals with a prior tuberculosis diagnosis had a positive result: 4/4 with recurrent TB and 2/40 (5%) with no recurrence. BAL-ELISPOT with a cut-off of >4,000 ESAT-6 or CFP 10 specific cells/1 million BAL lymphocytes detected all 4/4 individuals with recurrent tuberculosis accurately (Figure 2A, *P* <0.001). In addition, 38/40 individuals without recurrence of tuberculosis had a true negative test result and 2/40 individuals had a false positive test result leading to inappropriate treatment until negative culture results became available (Table 1, Figure 2).

Either a positive Xpert or a positive BAL-ELISPOT result was considered an indication to start tuberculosis treatment. Patient details of 8/44 patients with treatment indication are shown in Table 1. The different performances of the tests and their combinations are shown in Table 2.

**Table 2.** Performance of Different Testing and Result Interpretation Strategies.

CFP-10 or ESAT-6 cut-off	Positive Result	Negative Result	CC % of those tested	False +ve (unnecessary treatment) of non-TB	False -ve (missing treatment) of TB
Patients with a history of a previous TB disease and newly suspected recurrence of TB (*n*=44)					
Blood ELISPOT	36 (82%)	8 (18%)	12 (27%)	32 (80%)	0
BAL ELISPOT (Commercial cut-off)	17 (39%)	27 (61%)	31 (70%)	13 (33%)	0
>4000 BAL lymphocytes (ELISPOT positive)	6 (14%)	38 (86%)	42 (95%)	2 (5%)	0
>4000 BAL lymphocytes (ELISPOT positive) or positive Xpert	8 (18%)	36 (82%)	40 (91%)	4 (10%)	0
Xpert alone	3 (7%)	41 (93%)	39 (89%)	2 (5%)	3 (75%)

BAL: bronchoalveolar lavage; CC: correctly classified; NPV: negative predictive value; PPV: positive predicted value; TB: tuberculosis, CFP-10: culture filtrate protein 10, ESAT-6: Early secreted antigenic target 6kDa.

## DISCUSSION

In a cohort of patients with presumptive recurrent pulmonary tuberculosis we compared the diagnostic accuracy of Xpert and *M. tuberculosis*-specific ELISPOT from BAL cells. BAL-ELISPOT correctly detected all 4 patients with culture-confirmed recurrent tuberculosis and excluded recurrence with a specificity of 95% (in 38/40 patients) and was more accurate compared to Xpert for the diagnosis and exclusion of recurrent tuberculosis.

Among 44 individuals with a previous episode of tuberculosis, 9.1% (n=4) experienced a new episode of tuberculosis, in line with the estimated 7.7% recurrence according to the WHO data [16] and data from Germany, where almost 10% of patients had a previous tuberculosis diagnosis [17].

Recurrence of tuberculosis mostly occurs within the first 2 years after therapy [32] [33], a recent study reported an incidence rate of reactivation of 228/100,000 person years in the first 2 years and 57/100,000 person years in years 2-5 [34]. In this cohort, time between first episode and recurrence varied between 6 months and 60 years and 11/44 individuals had a previous episode less than 2 years prior.

Other studies [5, 11, 15] report that a substantial proportion of patients with undetectable acid-fast bacilli on sputum microscopy are missed if Xpert alone is used as a diagnostic tool. In our cohort of patients with presumptive recurrence of pulmonary tuberculosis, 3 out of the 4 patients with recurrent tuberculosis had a negative BAL-Xpert result which would have resulted in delayed treatment. Additionally, 2/40 patients (5%) with a previous history of tuberculosis had positive Xpert results despite negative *M. tuberculosis* cultures and — in the absence of disease recurrence — no need for antituberculous treatment. Both patients with a positive Xpert result, but no recurrent disease, had a previous tuberculosis episode less than 2 years prior to inclusion in the study.

Other studies have reported that 14.3% of patients with previous tuberculosis have persistently positive Xpert results without recurrent disease [23]. Positive Xpert results may persist for as long as 7 years following successful treatment [25]. This is explained by the persistence of mycobacterial DNA in the absence of viable bacteria [21].

While PBMC ELISPOT is not useful for the diagnosis of active tuberculosis as confirmed in this study, where 32/40 (80%) had a positive PBMC ELISPOT result, the detection of active tuberculosis can be improved by conducting ELISPOT tests on cells from the site of the infection [10–12, 35, 36]. Using the cut-off according to manufacturer's guidelines reveals low specificity for the BAL-ELISPOT. This problem has been addressed and different strategies for interpretation of test results in BAL have been evaluated [15, 30]. We proposed a cut-off by determining the number of lymphocytes in BAL fluid and using >4,000 *M. tuberculosis*-specific cells / 1 million lymphocytes to diagnose active tuberculosis, which improved sensitivity to 89% and specificity to 97% [15]. In contrast to the previous studies, where patients with recent history of tuberculosis (<2 years) had been excluded, the present study focused on the difficult-to-diagnose subgroup of patients with a history of tuberculosis. Of these 44 patients with a history of tuberculosis, all 4 patients with recurrent tuberculosis had more than 4,000 *M. tuberculosis*-specific cells / 1 million lymphocytes (sensitivity 100%). Of these 4 patients, 3 would have been missed by GeneXpert alone, ascertaining how important the combined strategy of BAL-GeneXpert and BAL-ELISPOT is for a sensitive and rapid diagnosis of active tuberculosis, and that in recurrent tuberculosis, BAL-ELISPOT might be more appropriate for the treatment decision than Xpert.

There were 38/40 patients who had a negative BAL-ELISPOT result or less than 4,000 *M. tuberculosis*-specific cells / 1 million lymphocytes (specificity 95%, Table 2). The median number of *M. tuberculosis*-specific cells/1 million lymphocytes was low (ESAT-6: 0 [IQR 0-621] and CFP10: 0 [IQR 0-383]) and very similar to that of patients without tuberculosis published earlier [15]. Among individuals with exclusion of recurrent TB, 2/40 (5%) have received unnecessary retreatment for tuberculosis because of a positive BAL-ELISPOT result with > 4,000 *M. tuberculosis*-specific cells / 1 million lymphocytes.

BAL-Xpert did not add to the sensitivity of the BAL-ELISPOT for the diagnosis of recurrent tuberculosis. In contrast, treatment decisions based on BAL-Xpert would have resulted in under-treatment of tuberculosis patients.

The main limitation of our study is the sample size of patients with recurrent tuberculosis. However, for the subgroup of patients with signs and symptoms of possible tuberculosis recurrence who underwent intensive diagnostic work-up including BAL, our study reports on the largest patient cohort to date to our knowledge.

While in a previous study on individuals with a first tuberculosis episode, the sequential approach of BAL-Xpert followed by BAL-ELISPOT had a sensitivity of 98.8% and a specificity of 97.6% [15], at present, we could show that in the diagnosis/exclusion of patients with recurrent tuberculosis BAL-ELISPOT was more accurate than BAL-Xpert and identified patients with high sensitivity and specificity. Another limitation is that only 1 HIV-positive patient could be included in the study and diagnosis of active tuberculosis by BAL-ELISPOT is challenging in people living with HIV [37].

Although BAL-ELISPOT is superior to Xpert for the diagnosis of recurrent paucibacillary tuberculosis, BAL-ELISPOT is more labor intensive than Xpert and requires the performance of flow cytometry on bronchial fluid lymphocytes. Due to the operational difficulties of performing bronchoscopies and BAL-ELISPOT in resource-limited settings, this test will likely only be applicable in high income countries.

## CONCLUSION

For individuals with presumed diagnosis of recurrent pulmonary TB in whom acid-fast bacilli or *M. tuberculosis*-specific DNA were not detectable on sputum and differential diagnosis required bronchoscopy, BAL-ELISPOT was more accurate for the diagnosis of active tuberculosis than Xpert with a sensitivity of 100% and a specificity of 95% in this study.

## SUPPLEMENTARY DATA

Supplementary materials are available at the *Pathogens and Immunity* website. Supplementary data may be provided by the authors to benefit the reader. Supplementary data are not copyedited and are the sole responsibility of the authors. Questions or comments related to supplementary materials should be addressed to the corresponding author.
