# Supplementary material for: Rapid Diagnosis of Recurrent Paucibacillary Tuberculosis
Source: Pathog Immun. 2023 Apr 19;7(2):189–202. doi: 10.20411/pai.v7i2.565 (PMC10189871; doi:10.20411/pai.v7i2.565)
Supplement: Supplemental Figure 1 [file pai-7-189-s01.pdf]

PATHOGENS AND IMMUNITY
IN ASSOCIATION WITH CASE WESTERN RESERVE UNIVERSITY AND THE RICHARD J. FASENMYER FOUNDATION

SUPPLEMENTARY FIGURE 1

Supplementary Figure 1. Flow cytometric analysis of the percentage of lymphocyte in bronchoalveolar lavage cells. A representative contour plot from a single patient is shown, cells were acquired on a FACSCalibur flow cytometer (BD Bioscience, Heidelberg, Germany). Gating strategy used forward-scatter-height (FSC) versus side scatter height parameters. Region 1 was gated on monocytes/macrophages (R1, gated number of cells given in % of total) and Region 2 was gated on BAL-lymphocytes (R2, gated number of cells given in % of total) in Region statistics underneath. The respective percentage of BAL-lymphocytes out of the population of BAL-monocyte/macrophages (R1) and BAL-lymphocytes (R2) was used to extrapolate SFCs per 250,000 cells per well to 1 million lymphocytes in BAL.

Calculation: (SFC /250.000 BALCs X 4) x (G1+G2 in %) / (G2 in %) = SFC / 1 million BAL-lymphocytes

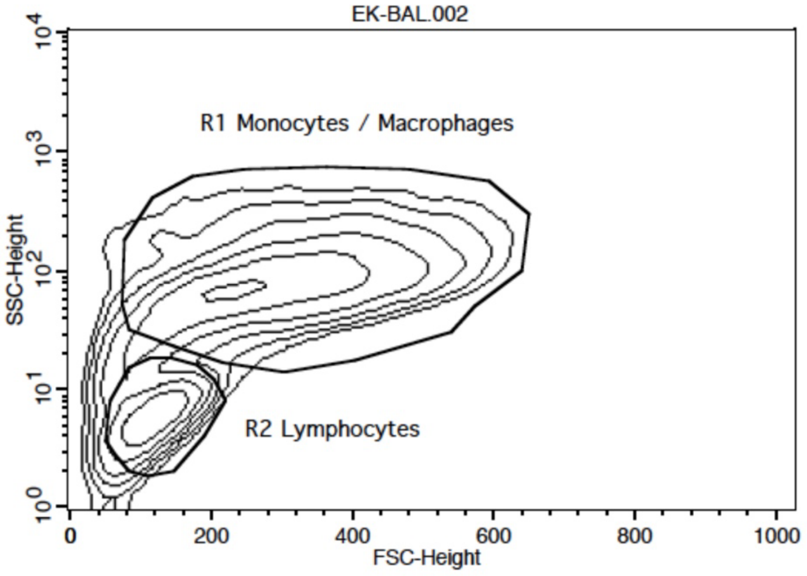

Region Statistics

Acquisition Date: 18-Mar-20
Total Events: 46028
X Parameter: FSC-Height (Linear)
Y Parameter: SSC-Height (Log)

Region	% Total
R1 Monocytes / Macrophages	63.45
R2 Lymphocytes	29.35

Jafari C, Olaru ID, Daduna F, Lange C, Kalsdorf B. Rapid Diagnosis of Recurrent Paucibacillary Tuberculosis. *Pathogens and Immunity.* 2023;7(2):189-202. doi: 10.20411/pai.v7i2.565
